# Tweeting links to cochrane schizophrenia group reviews: a randomised controlled trial

**DOI:** 10.1186/1745-6215-16-S2-P165

**Published:** 2015-11-16

**Authors:** Clive Adams, Mahesh Jayaram, Angelique Bodart, Stephanie Sampson, Sai Zhao, Alan Montgomery

**Affiliations:** 1 University of Nottingham, Nottingham, UK; 2 University of Melbourne, Melbourne, Australia; 3 Systematic Review Solutions, Yantai, China

## Introduction

Abstracts and plain language summaries of Cochrane reviews are freely available on the Cochrane website. The aim of this study was to investigate the effect of tweeting review precis on web traffic to Schizophrenia Group reviews.

## Methods

In an individually randomised controlled parallel group superiority trial, we allocated Cochrane Schizophrenia Group reviews with an abstract and plain language summary web page to either intervention or control using a computer generated random sequence. Reviews in the intervention arm had three randomly ordered messages of up to 140 characters, each containing a short URL to the freely accessible summary page, sent via Twitter and Weibo at specific times on one single day. This was compared with no messaging. The primary outcome was web page visits during a one week follow up period, collected using Google Analytics.

## Results

Eighty-five reviews were allocated to each arm, and 100% of outcome data were collected and analysed. Intervention and control reviews received a total of 1162 and 449 visits respectively (IRR 2.7, 95% CI 2.2 to 3.3). Fewer intervention reviews had single page only visits (16% vs 31%, OR 0.41, 0.19 to 0.88) and users spent more time viewing intervention reviews (geometric mean 76 vs 31, ratio 2.5, 1.3 to 4.6). Other secondary metrics of web activity all showed strong evidence in favour of the intervention.

## Conclusion

Tweeting in this limited area of health care increases ‘product placement’ of evidence with the potential for that to influence care.

## Trial Registration number

ISRCTN84658943

